# Supplementary material for: High-resolution line-scan Brillouin microscopy for live imaging of mechanical properties during embryo development
Source: Nat Methods. 2023 Mar 30;20(5):755–60. doi: 10.1038/s41592-023-01822-1 (PMC10172129; doi:10.1038/s41592-023-01822-1)
Supplement: Supplementary file 1 — Supplementary Note 1, Supplementary Table 1, Supplementary Fig. 1 and Supplementary References. [file 41592_2023_1822_MOESM1_ESM.pdf]

nature methods

Article https://doi.org/10.1038/s41592-023-01822-1

High-resolution line-scan Brillouin microscopy for live imaging of mechanical properties during embryo development

In the format provided by the authors and unedited

SUPPLEMENTARY INFORMATION

Table of Contents

SUPPLEMENTARY NOTES..2
SUPPLEMENTARY TABLE 7
SUPPLEMENTARY FIGURES 8
SUPPLEMENTARY REFERENCES..9

SUPPLEMENTARY NOTE

SI Note 1: LSBM performance and comparison

This Note discusses additional technical details of our LSBM system, such as the performance and various trade-offs between our two modalities (O-LSBM and E-LSBM), the fundamental limits to spatial and mechanical resolution of our method, and on how fluorescence SPIM can assist in the interpretation of Brillouin spectral data.

Comparison of spatial resolution in O-LSBM vs. E-LSBM

The spatial resolution in Brillouin microscopy is determined by an interplay between the propagation of light and acoustic waves (phonons) within the imaged voxel (defined by the optical point-spread function, PSF). For (complex) biological samples the propagation of the probed phonons is not trivial, with the consequence that it is not possible to define a spatial resolution *a priori*, without a knowledge of the sample. An in-depth discussion about this issue, supported by experimental data, can be found in Ref. [1].

In this Supplementary Note we will only introduce the key concepts necessary to compare the achievable spatial resolution in the O-LSBM and E-LSBM geometries.

The ultimate limit for the spatial resolution is the extent of the volume probed by the light, i.e. the optical PSF, which for our LSBM is reported in Fig. 1e and Ext. Data Fig. 4a-b. In E-LSBM, the PSF is fully determined by the NA of the objective. This implies that the z-resolution is significantly worse than the xy resolution (similarly to point-scanning confocal microscopy). Note that, differently from a point confocal, the x and y resolution in the E-LSBM are slightly different because the slit is acting as a confocal pinhole only along the y direction. The x/y resolution of O-LSBM is similar to the one of the E-LSBM since it is mainly determined by the detection objective. On the contrary, the z resolution of the O-LSBM is significantly better (~3x) than the one of the E-LSBM thanks to the optical sectioning of the illumination line. In fact, when the thickness of the illumination line is smaller than the z resolution of the detection objective, the z extent of the PSF coincides with the thickness of the illumination line. The latter can be varied by changing the effective NA of the illumination objective (i.e. changing the beam size on the back focal aperture of the objective). We designed the thickness of the illumination line to be ~1μm, that is the typical mechanical resolution achievable in a biological sample.

Furthermore, we note that the mechanical properties are probed by the acoustic waves and not by the illumination light itself. Therefore, the spatial extent of the acoustic waves, L_a, sets an additional limit to the mechanical resolution. L_a intrinsically depends on the material under investigation and can be calculated from the measured Brillouin spectrum, being proportional to the inverse of the linewidth. The linewidth of pure water, in epi-detection at 780nm, is $\Gamma_B = 308\text{MHz}$ (FWHM) [2]. From there the 1/e lifetime of the phonon can be calculated as $(2\pi \cdot \Gamma_B)^{-1} = 0.52\text{ns}$. The velocity of acoustic waves in water is V = 1480m/s, it thus follows that $L_a = V \cdot (2\pi \cdot \Gamma_B)^{-1} = 0.75\mu m$. Due to the different scattering geometry of the O-LSBM and the E-LSBM both the direction and the wavelength of the probed phonon differs. Specifically, the wavelength of the phonon in the O-LSBM is a factor $\sqrt{2}$ (i.e. sin(90/2)) longer than in the E-LSBM. Consequently, due to its quadratic dependence on the wavelength in water [3], L_a is doubled in O-LSBM (~1.5um) compared to the E-LSBM . Brillouin spectra from biological matter typically feature a linewidth larger than (but comparable to) the one of water, thus making ~1μm a good target resolution. We note that small structures with very distinct mechanical properties could alter the propagation of acoustic waves. In that case structures smaller than ~1μm can in principle be resolved (see e.g. Ref. [4]). But these are very specific cases where a careful optimization of the imaging parameters is essential (usually at the cost of reduced speed and acquired volume). In our

work, in contrast we designed the LSBM to enable fast volume imaging of light-sensitive samples over long time periods.

We conclude by briefly commenting on the link between spectral resolution and mechanical resolution. If different mechanical compartments are present within the PSF of the microscope that are larger than L_a, the Brillouin spectrum will display different peaks that can be resolved in principle (usually few hundreds of MHz apart) [5,6]. Since our LSBM is based on a VIPA etalon, it features a relatively poor spectral resolution (~500MHz, see Ext. Data Fig. 5d), which especially in the O-LSBM, is affected by the broadening of the linewidth due to the use of high NA optics [7]. Therefore, it is challenging to resolve closely spaced Brillouin peaks with LSBM. We note, however, that even though resolving different peaks provides additional information about the heterogeneity of the sample within the probed volume (PSF), this information is not improving the spatial resolution as it is not possible to reassign the different components to their corresponding spatial location.

Efficiency in O-LSBM vs. E-LSBM

In this subsection we calculate and compare the collection efficiency of the O-LSBM vs. E-LSBM. We coarsely follow the derivation in Ref. [8] but adapted to our LSBM implementation. In the approximation of constant intensity within the interaction volume, the collected scattering power is $P_s = I_{ill} \cdot V \cdot \Omega \cdot R$ where I_{ill} is the illumination intensity [W/m^2], V is the interaction volume [m^3], Ω the collected solid angle (that is given by $\Omega = 4\pi\left(\frac{NA}{n}\right)^2$) when limited by the NA of the detection objective in a medium having refractive index n, R is the Brillouin scattering coefficient [m^{-1}], which can be considered constant here. We note that the previous formula does not take into account the rejection of out of focus light due to confocal detection but such a correction is relevant only if the illumination line in the O-LSBM would be thicker than the z-resolution of the detection objective, which is not the case in our experimental setting. Next, we apply this formula to calculate the Brillouin signal collected from each point along the illumination line in the O-LSBM and E-LSBM configuration; we will add a subscript O and E to the quantities referring to the O-LSBM and E-LSBM configuration respectively. Both O-LSBM and E-LSBM share the same detection objective so $\Omega_O = \Omega_E$. Following the same convention for the axes as in Fig. 1c and 1d, the interaction volume that corresponds to a single pixel on the camera can be approximated as $V = L_x/N \cdot l_y \cdot l_z$ where $L_x \approx 200um$ is the extension of the illumination light, $N \approx 280$ is the number of pixels on the camera that cover the full extent of the illumination light, and l_y and l_z are the extent of the illuminated volume in y and z. In the O-LSBM l_y and l_z are determined by the illumination NA. In the E-LSBM l_y and l_z are determined by the NA of the detection objective.
In our experiments we used similar illumination power for the O-LSBM and E-LSBM ($P_{ill,O} \approx P_{ill,E}$ ≈20mW). For the E-LSBM $I_{ill,E} \approx \frac{P_{ill}}{L_x \cdot l_{y,E}}$. For the O-LSBM, the intensity of the static beam would be $\frac{P_{ill}}{l_{y,O} \cdot l_{z,O}}$ but the tunable lens "spreads" the intensity that extends over a length of $x_{R,O} = \pi/ln(2) \cdot n \cdot l_{z,O}^2/\lambda$ (Rayleigh range, n is the refractive index of the immersion medium) over a length L_x, therefore $I_{ill,O} \approx \frac{P_{ill}}{l_{y,O} \cdot l_{z,O}} \cdot \frac{x_{R,O}}{L_x}$. Using the values reported in Fig. 1e $x_{R,O} = 4.53 \cdot 1.33 \cdot \frac{1.23^2}{0.78} = 11.69\mu m$
If we then calculate the ratio of collected scattering power in the two geometries we obtain:

$$\frac{P_{s,O}}{P_{s,E}} = \frac{I_{ill,O} \cdot V_O \cdot \Omega \cdot R}{I_{ill,E} \cdot V_E \cdot \Omega \cdot R} = \frac{P_{ill}}{l_{y,O} \cdot l_{z,O}} \cdot \frac{x_{R,O}}{L_x} \cdot \frac{L_x \cdot l_{y,E}}{P_{ill}} \cdot \frac{L_x/N \cdot l_{y,O} \cdot l_{z,O}}{L_x/N \cdot l_{y,E} \cdot l_{z,E}} = \frac{x_{R,O}}{l_{z,E}} = \frac{8.79}{3.90} \approx 3$$

Despite the several approximations used in this calculation (i.e. assuming the intensity constant within the interaction volume and using the formulas for Gaussian beams), it shows that the Brillouin signal collected in the two geometries is comparable, consistent with our experimental observations.

Precision in O-LSBM vs. E-LSBM

In the assumption that the Brillouin spectrum is composed of a single peak, the Brillouin shift (i.e. the position of the peak) can be determined with a precision that is much higher than the spectral resolution of the spectrometer. In fact, when the detection is limited by shot noise, the precision scales as the width of the peak divided by the square root of the number of detected photons [9].

$$\sqrt{< \nu_B^2 >} = \Gamma_B / \sqrt{N} \quad (1)$$

In this regime the precision can be arbitrarily increased at the expense of higher illumination energy, that is the product of illumination power times exposure time (Ext. Data Fig. 4d). From Ext. Data Fig. 4d it can be observed that the precision in O-LSBM and E-LSBM at the same illumination energy differs. This is due to a difference in both terms in Eq. (1). As calculated in the previous note, given the same optical power on the sample the number of collected photons is different in O-LSBM and E-LSBM by a factor ~3. But the biggest contribution is the difference in linewidth; in fact the broadening due to the use of high NA optics is much more pronounced at a 90deg scattering angle [7] resulting in a linewidth that is ~2.5 folds broader in the O-LSBM compared to the E-LSBM. Inputting this value in Eq. (1), we find that the O-LSBM is expected to have a precision that is $\sim 2.5/\sqrt{3} \sim 1.44$ folds worse than E-LSBM. That is consistent with the experimentally measured values (Fig. 1f): the precision of the O-LSBM divided by the precision of the E-LSBM gives 19.33/12.79=1.51. Indeed, the lower precision of the O-LSBM might affect the ability to visually identify structures if the difference between their Brillouin shift and the surrounding pixels falls below the precision. But, if the aim of the experiment is to measure the Brillouin shift of some specific structure, one can segment the structure of interest from the fluorescence channel and average the pixels belonging to it, thus gaining more statistical power to actually distinguish it. This highlights the usefulness and power of the additional fluorescence SPIM modality.

Fluorescence SPIM modality assists quantification of Brillouin images

As outlined in the main text and Method section, we added a custom-built concurrent selective plane illumination microscopy (SPIM) fluorescence imaging modality to our LSBM platform. This allows for fluorescence guided Brillouin image analysis in 3D, and in particular, it provides capability along the following:

(1) It assists with identifying fluorescently labelled structures of interest (cellular compartment, cell or tissue types) in 3D, so that the Brillouin imaging pixels belonging to these structures can be properly segmented, analyzed and quantified. This enables quantification and thus measurement of e.g. the ‘average’ mechanics of a particular region such as a cell or tissue type, which could otherwise be difficult in case of insufficient mechanical contrast. Such analysis can also improve statistical power as outlined in the previous section.

(2) Using fluorescence as a guide for the image analysis further reduces bias (i.e. segmenting the ROI from the Brillouin contrast could be misleading) and enables correlating and assigning mechanical properties to different (molecular) constituents or tissue regions, hence aiding in data interpretation.

(3) Before LSBM imaging, fluorescence SPIM can provide guidance to the experimenter in selecting the desired spatial area and/or time-point to be imaged.

(4) Fluorescence SPIM images can further provide information about the proper development, stage and health of the sample, and thus provide useful feedback for the experimenter when selecting and validating samples for a given experiment.

In our experiments, SPIM imaging is performed only once for every Brillouin time-lapse volume, and the excitation powers are comparatively very low (<~0.1mW). Therefore, phototoxic effects are unlikely, although we point out that the (low) additional photo-burden needs to be taken into account when choosing imaging parameters for very sensitive sample types. Finally, we note that fluorescence SPIM is not strictly necessary for LSBM imaging, but becomes an additional asset for quantitative, spatially resolved analysis of Brillouin images in 3D, and thus in their biophysical interpretation.

Acquisition time of the LSBM

The LSBM has the ability to acquire up to >~200 spectra along the illumination line in parallel. It follows that, with an exposure time of 100ms, the effective acquisition time per pixel is less than 1ms. It is important to note though that the light from each pixel is still integrated over 100ms, which is thus the fastest dynamics that could be caught when imaging a single line without scanning. Additionally, the speed improvement is proportional to the size of the sample in the direction of the illumination line (i.e. the longer the sample the shorter the effective acquisition time per pixel). It is thus beneficial to align the longest axis of the sample to the illumination line.

The current spectrometer framerate (~10Hz) is lower than what the EM-CCD would allow (~100Hz for the size of the sensor we are using) and it would thus in principle be possible to decrease the exposure time of the camera while increasing the optical power to keep the same SNR. For our particular applications and experiments, we optimised the system for low optical power while maintaining a relatively high precision of ~10MHz, comparable with a state-of-the-art VIPA-based Brillouin spectrometer [10]. For this effective pixel times of <1ms were achievable and sufficient for the biological experiments, hence we chose an overall exposure time of 100ms for the EMCCD camera. However, for further imaging speed optimizations also the dead time of ~50ms between subsequent camera acquisitions need to be accounted for. The dead time is mainly determined by the movement of the stage to the subsequent scan position, due to the communication delays of LabView and the response time of the controller of the stage. In the future, this could be significantly reduced by directly hardware-triggering the stage movement.

Finally, we note that it is in principle possible to speed up data acquisition by only imaging individual lines (or multiple points with a confocal Brillouin microscope) in the sample instead of 3D volume imaging (potentially guided by fluorescence). While this could be a feasible strategy for immobilised or slowly developing specimens, it would likely yield incomplete information on dynamic events such as morphogenesis, which involves rapid, three-dimensional shape change of cells, tissues, organs and organisms as whole.

Comparison between point-scanning confocal and LSBM

In this subsection we aim to compare the performance of LSBM with a state-of-the-art point-scanning confocal Brillouin microscope (CBM). As outlined in the first section of the note, the spatial resolution is fundamentally limited by the propagation of the phonon (limit shared by both the LSBM and CBM) and, additionally, by the optical resolution of the microscope. For the latter we choose an intermediate-high NA compared to other studies [10] with the aim of studying small organisms *in toto* with subcellular resolution. We note that LSBM lacks the flexibility of easily changing the objective lens, but in principle, inverted V-configuration featuring higher NA and thus optical resolution can be designed [11]. In our work, however,

we optimised the resolution to be ~1µm, thus matching the phonon's attenuation length expected in our samples (see section above).

Another important parameter to consider in Brillouin microscopy is the precision: in the E-LSBM we achieve a precision of ~13MHz, which is comparable to a state-of-the-art VIPA-based CBMs (see Ref. [10]). We note that we could achieve this precision with significantly lower energy delivered to the sample compared to CBMs. In fact, Schlüßler et al. [12] demonstrated the use of a 780nm CBM for imaging of small organisms (zebrafish larva), which can be considered similar to the samples studied in our work. This implementation achieved a precision of 10MHz with a power of 10mW and exposure time of 500ms, thus requiring an illumination energy of 5mJ/px. In contrast, our total illumination energy is 2mJ (20mW, 100ms) but distributed over ~200 pixels, therefore resulting in an illumination energy of only ~0.01mJ/px; that is ~500x lower than Ref. [12]. Since the photodamage is proportional to the light dosage, the 500x lower illumination energy per pixel of the E-LSBM compared to a CBM, corresponds to an 500 times lower photodamage. The O-LSBM has slightly worse precision than the E-LSBM, given the same illumination power (Fig. 1f), but it has the additional advantage of avoiding the out of focus illumination, that corresponds to an additional reduction of photodamage that roughly scales with the number of acquired z planes.

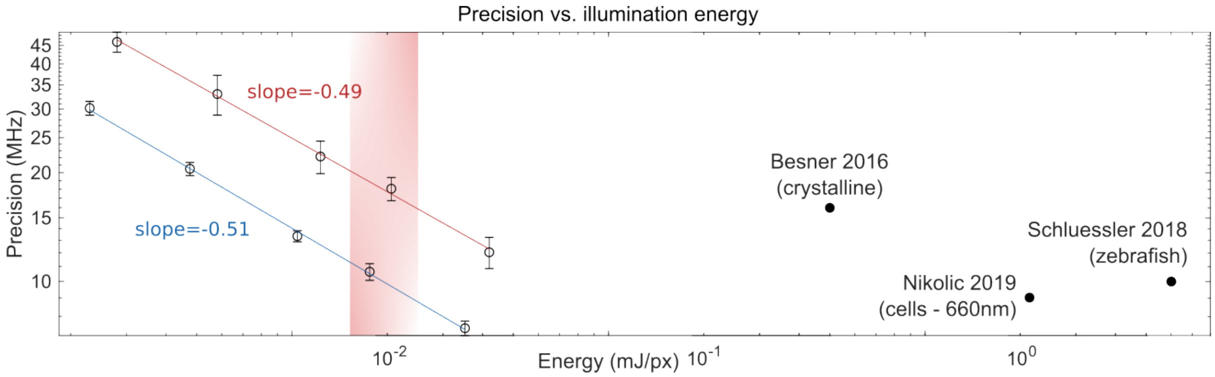

SI Note Fig. 1: Precision vs. illumination energy for the orthogonal (red) and epi (blue) line geometries and comparable confocal Brillouin microscopy implementations. The fit shows a square root dependence, as expected in shot noise limited conditions. The shaded red region indicates the typical imaging conditions used in the experiments. Error bars represent S.D.

Effect of refraction on the LSBM signal

The heterogeneity of refractive index within the sample and the difference in refractive index between the medium and the sample can cause refraction of both the illumination and detected light. In fluorescence lightsheet microscopy refraction causes a loss of signal and a deterioration of resolution [13]. Additionally in Brillouin microscopy refraction can cause a change in the scattering angle (i.e. the angle between the incident and scattered wavevector), which in turns affect the measured Brillouin shift (the Brillouin shift is proportional to $\sin(\theta/2)$). Some samples can be particularly affected by this issue, e.g. Drosophila embryos whose optical properties are notoriously heterogeneous. Our solution to this problem was the introduction of the E-LSBM. Being based on an epi-detection scheme, the scattered light follows the same (reversed) optical path as the illumination. Therefore, the scattering angle θ is always 180deg (at least at a first approximation, when low NA is considered). Additionally, at 180deg the change in Brillouin shift due to a change in the scattering angle θ (i.e. the derivative of $sin(\theta/2)$) is minimised. Note that other approaches, like the use of a dual illumination [14] can be used to mitigate the effect of refraction.

SUPPLEMENTARY TABLE

SI Table 1: LSBM acquisition parameters.
This Table summarises the experimental parameters used during LSBM imaging. Note that all experiments used the same sampling (pixel size) along x, i.e. 0.71µm (see Ext. Data Fig. 4c). SV, Supplementary Video.

Fig.	modality	y step (µm)	z step (µm)	Imaged volume (um)	Overall imaging time	Acquisition time	Time resolution	Optical power on the sample (mW)
2c and SV1	E-LSBM	1.5	1.5	~22×180×71	~41min	129s	129s	<~15
2f and SV2	E-LSBM	1.5	2.5	~83×183×43	~33min	124s	124s	<~20
3b	O-LSBM	2.5	2.5	~165x186x172	N/A	~17min	~17min	<~15
3g and SV3-4	E-LSBM	2.5	3	~192x190x111	~14h	~10min	~29min	<~18
S8 and SV5-6(first 26h)	O-LSBM	1.5	1.5	~92x120x100	~26h	~13min	77 min	<~15
S8 and SV5-6 (later)	O-LSBM	1.5	1.5	~111x131x121	~20h	~16min	92min	<~15
S11a	E-LSBM	2.3	2.3	~152x171x121x140	~1h	~11min	~11min	<~16
S11b	E-LSBM	2.3	2.3	~152x161x121x140	~45min	~11min	~11min	<~16

SUPPLEMENTARY FIGURES

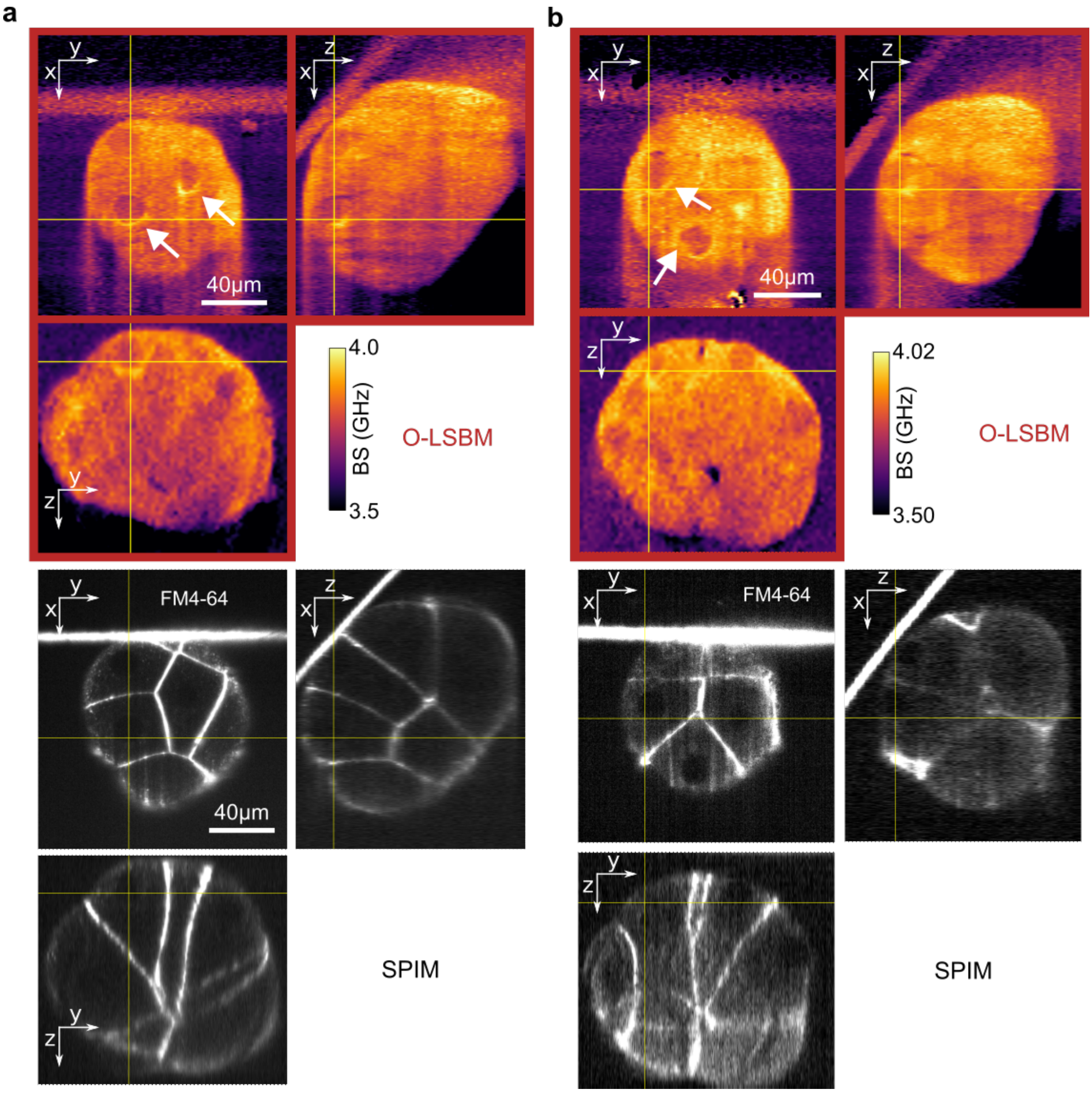

SI Fig. 1: Peri-nuclear high Brillouin shift in Phallusia 16-cell state embryos. (a-b) Orthogonal views of the Brillouin shift map (top) and corresponding SPIM volume (bottom) of two representative 16-cells stage Phallusia embryos. The white arrows indicate a high Brillouin shift surrounding the nucleus. In total, we found a high BS signal surrounding at least one nucleus in 73% of imaged embryos (n=15) at the early-to-late 16 cells transition, which suggests this to be a cell cycle stage specific structure.

SUPPLEMENTARY REFERENCES

1. S. Caponi, D. Fioretto, and M. Mattarelli, "On the actual spatial resolution of Brillouin Imaging," Opt. Lett. **45**, 1063–1066 (2020).
2. I. Remer, R. Shaashoua, N. Shemesh, A. Ben-Zvi, and A. Bilenca, "High-sensitivity and high-specificity biomechanical imaging by stimulated Brillouin scattering microscopy," Nat. Methods **17**, 913–916 (2020).
3. S. L. Garrett, "Attenuation of Sound," in (2020), pp. 673–698.
4. C. Bevilacqua, H. Sánchez-Iranzo, D. Richter, A. Diz-Muñoz, and R. Prevedel, "Imaging mechanical properties of sub-micron ECM in live zebrafish using Brillouin microscopy," Biomed. Opt. Express **10**, 1420 (2019).
5. S. Mattana, M. Mattarelli, L. Urbanelli, K. Sagini, C. Emiliani, M. D. Serra, D. Fioretto, and S. Caponi, "Non-contact mechanical and chemical analysis of single living cells by microspectroscopic techniques," Light Sci. \& Appl. **7**, 17139 (2018).
6. R. Prevedel, A. Diz-Muñoz, G. Ruocco, and G. Antonacci, "Brillouin microscopy: an emerging tool for mechanobiology," Nat. Methods **16**, 969–977 (2019).
7. G. Antonacci, M. R. Foreman, C. Paterson, and P. Török, "Spectral broadening in Brillouin imaging," Appl. Phys. Lett. **103**, 5–8 (2013).
8. J. Zhang, A. Fiore, S.-H. Yun, H. Kim, and G. Scarcelli, "Line-scanning Brillouin microscopy for rapid non-invasive mechanical imaging," Sci. Rep. **6**, 35398 (2016).
9. G. Zanini and G. Scarcelli, "Localization-assisted stimulated Brillouin scattering spectroscopy," APL Photonics **7**, 056101 (2022).
10. G. Antonacci, T. Beck, A. Bilenca, J. Czarske, K. Elsayad, J. Guck, K. Kim, B. Krug, F. Palombo, R. Prevedel, and G. Scarcelli, "Recent progress and current opinions in Brillouin microscopy for life science applications," Biophys. Rev. **12**, 615–624 (2020).
11. P. Strnad, S. Gunther, J. Reichmann, U. Krzic, B. Balazs, G. de Medeiros, N. Norlin, T. Hiiragi, L. Hufnagel, and J. Ellenberg, "Inverted light-sheet microscope for imaging mouse pre-implantation development," Nat. Methods **13**, 139–142 (2015).
12. R. Schlüßler, S. Möllmert, S. Abuhattum, G. Cojoc, P. Müller, K. Kim, C. Möckel, C. Zimmermann, J. Czarske, and J. Guck, "Mechanical Mapping of Spinal Cord Growth and Repair in Living Zebrafish Larvae by Brillouin Imaging.," Biophys. J. **115**, 911–923 (2018).
13. L. A. Royer, W. C. Lemon, R. K. Chhetri, Y. Wan, M. Coleman, E. W. Myers, and P. J. Keller, "Adaptive light-sheet microscopy for long-term, high-resolution imaging in living organisms," Nat. Biotechnol. **34**, 1267–1278 (2016).
14. J. Zhang, M. Nikolic, K. Tanner, and G. Scarcelli, "Rapid biomechanical imaging at low irradiation level via dual line-scanning Brillouin microscopy," bioRxiv 2022.04.25.489096 (2022).
